# Supplementary material for: Our perception may not be reality: A longitudinal study of the relationship between perceived and actual change in smoking behavior
Source: PLoS One. 2024 Apr 1;19(4):e0301515. doi: 10.1371/journal.pone.0301515 (PMC10984459; doi:10.1371/journal.pone.0301515)
Supplement: S1 Table — TEMPO cohort (n = 134), GEE model (OR, 95% CI). (DOCX) [file pone.0301515.s001.docx]

| S1 Table. Socio-demographic characteristics of the study population and unadjusted associations with a mismatched tobacco use assessment. TEMPO cohort (n=134), GEE model (OR, 95% CI). | | | | | |
| --- | --- | --- | --- | --- | --- |
|  | |  | n (%) | OR [95% CI] | p-value |
| Change in the number of cigarettes (25%) * | | |  |  |  |
| Yes, increased | |  | 45 (32.1%) | **2.56 [1.40;4.68]** | <0.0001 |
| Yes, decreased | |  | 39 (28.9%) | **5.46 [3.10;9.62]** | 0.0154 |
| No | |  | 50 (38.9%) | Ref |  |
| Sex | | | | | |
|  | Female |  | 87 (60.1%) | 0. 80 [0.50;1.24] | 0.3092 |
|  | Male |  | 47 (39.9%) | Ref |  |
| Age | | | | | |
|  | Age (continuous), mean (std) |  | 39.8 (0.35) | 1.00 [0.94;1.07] | 0.9901 |
| Marital status | | | | | |
|  | Single, divorced or widowed | | 29 (22.3%) | **1.53 [0.99;2.35]** | 0.0520 |
|  | Married, civil union or in a relationship | | 105 (77.7%) | Ref |  |
| Educational level | | | | | |
|  | Bac+2 or less |  | 45 (37.7%) | 1.10 [0.67;1.78] | 0.7107 |
|  | Bac+3 or more |  | 89 (62.3%) | Ref |  |
| Employment status | | | | | |
|  | Unemployed |  | 12 (10.2%) | 0.93 [0.49;1.79] | 0.8358 |
|  | Employed |  | 122 (89.8%) | Ref |  |
| Type of employment | | | | | |
|  | Unstable |  | 19 (15.1%) | **1.58 [0.88;2.84]** | 0.1278 |
|  | Stable |  | 115 (84.9%) | Ref |  |
| Household income | | | | | |
|  | 2500 euros or less |  | 34 (26.9%) | 1.10 [0.67;1.79] | 0.7144 |
|  | 2501 euros or more |  | 100 (73.1%) | Ref |  |
| Symptoms of depression in 2020 * | | | | | |
|  | Yes |  | 28 (21.9%) | **1.33 [0.83;2.11]** | 0.2330 |
|  | No |  | 106 (78.1%) | Ref |  |
| Smoking status in 2018 | |  |  |  |  |
|  | Daily smoker | | 95 (72.6%) | 0.83 [0.54;1.27] | 0.3876 |
|  | Non-daily smoker (including non-smokers and occasional smokers) | | 39 (27.4%) | Ref |  |
| * Changing each wave; the distribution reflects the first time answering a TEMPO COVID-19 questionnaire. | | | | | |
